# Prescriptions and Dispensing

**Published:** 1907-10-05

**Authors:** 


### THERAPEUTICS.

### PRESCRIPTIONS AND DISPENSING.

### INTRODUCTION.

THE medical student of to-day has many advantages over the medical student of fifty years ago, but he has the very big disadvantage in that at no period of his medical education does he come into anything like close contact with the difficulties there may be in dispensing one drug in mixture with another. He is often quite ignorant, long after he is qualified, of the taste, appearance, and smell of any particular dose that he may be ordering; many mistakes in prescription writing would speedily be corrected if, in his student days, it had been necessary for him to go through a course of dispensing his own and other people's prescriptions, as was the case in the old days of apprenticeship.

The practitioner who dispenses his own medicines soon realises this deficiency in the present medical curriculum, and corrects it for himself by practical work in his own dispensary; he also realises very soon another important point about drugs—namely, their relative cost. It is certain that much expense might be saved to general hospitals if the out-patient officers and house physicians and house surgeons had some practical experience as to what drugs are expensive, and what are cheap: in many cases the less expensive remedy might be just as efficacious as the more costly; but the latter is apt to be used when the prescriber has not to pay the bill.

Many practitioners nowadays, however, do not dispense at all, and unless they make special efforts to do so they may never know whether the medicine they are giving to a particular patient is a nice one or a nasty. There are obvious objections to prescribing drugs in tablet form; in the first place, some tablets pass through with the motions undissolved; in the next place, it is difficult to make small but important changes in the dosage when the tablet is prescribed; tablets are so easily obtained by the patient that once prescribed they may be taken for subsequent ailments in which they may do more harm than good; it is impossible to prescribe certain drugs, digitalis for example, in their most efficient form when tablets are used; and the efficacy of a mixture often depends upon its corrective ingredient as much as upon its most active principle, and it is not easy to make tablets which contain just the right proportions of each individual drug required for the most successful treatment of one particular case. Tablets may be right enough in the treatment of a particular disease, but it is very rarely that the physician can treat a disease as a disease.

The difficulties that a dispensing chemist may be put to in trying to make up a prescription sent him by a doctor inexperienced in prescribing are often very great, and this is a slur upon the profession. It is the fault of the present curriculum rather than of the individual, but one has only to look at the *Pharmaceutical Journal* correspondence columns to see such letters as the following:—

"*A Dispensing Difficulty.*

"How should this be sent out?

℞ Zinc sulphocarb. ... ... ... ... ʒijss.

Borax ... ... ... ... ... ʒijss.

Sod. Bic. ... ... ... ... ... ʒj.

Ac. Carbolic ... ... ... ... ʒss.

Aq. ... ... ... ... ... ad ℥x.

M. ft. lot. dilutio. To be used as directed, once a day.

"Is it the idea of the doctor to obtain a precipitate of $ZnCO_3$, which comes down if made as written, leaving a straw-coloured solution on the top? Or ought the $NaHCO_3$ to be left out? It was impossible to communicate with the doctor. What should be done?"

The doctor who has no opportunity to dispense can learn much from the dispensing chemist, and it would be a most wise thing on the medical student's part if he were to visit his hospital dispensary from time to time and see the methods there employed. It is very difficult to learn about these things from books; but the following are a series of articles upon the subject, based by kind permission upon dispensing difficulties that have been discussed from time to time in the *Pharmaceutical Journal.*

### GENERAL PRINCIPLES.

The preparation of medicines falls naturally into two divisions; in the first the drugs in their original condition are subjected to various processes by which the valuable constituents are brought into the form of tinctures, infusions, syrups, powders, and so forth, such processes constituting galenical pharmacy; in the second, the galenical preparations are themselves compounded together or with other substances, the product being intended for the use of some individual patient. It is this second division of the preparation of medicines which constitutes dispensing.

The business of the dispenser, whether medical man or chemist, is to produce the required medicine in such a form that every dose shall contain a uniform proportion of each of the ingredients; that the activity of one drug shall not be diminished by improper mixing with another of a different nature; that a soluble substance shall not be rendered practically insoluble by being massed into a pill, for example, by the aid of something which will hinder its dissolution; and that the medicine shall be presented to the patient in the most pleasing and palatable form consistent with its nature. The general nature of chemical action must be known, while familiarity with the principles of solution, and the general effects of heat on substances, the nature of tinctures, extracts, and other galenical preparations, and of resins, alkaloids, glucosides, and other active principles is essential. It should be the constant aim of the student to understand the reason *why* a particular procedure is followed in any given case, and in the present articles the principles involved in the methods described will be especially insisted on.

We may summarise these preliminary remarks by saying that good dispensing, and consequently good prescription writing also, require the application of common sense to a knowledge of the substances to be dealt with, and of the end to be attained.

(*To be continued.*)

